# Tunable Bragg polaritons and nonlinear emission from a hybrid metal-unfolded ZnSe-based microcavity

**DOI:** 10.1038/s41598-017-00878-2

**Published:** 2017-04-10

**Authors:** SK. Shaid-Ur Rahman, Thorsten Klein, Jürgen Gutowski, Sebastian Klembt, Kathrin Sebald

**Affiliations:** 1 Semiconductor Optics, Institute of Solid State Physics, University of Bremen, Bremen, 28334 Germany; 2 Semiconductor Epitaxy, Institute of Solid State Physics, University of Bremen, Bremen, 28334 Germany; 3 BIAS, Bremer Institut für angewandte Strahltechnik GmbH, Klagenfurter Str. 2, 28359 Bremen, Germany; 4 Technische Physik, Universität Würzburg, Am Hubland, D-97074 Würzburg Germany

## Abstract

Strong light-matter interaction in Bragg structures possesses several advantages over conventional microcavity system. These structures provide an opportunity to incorporate a large number of quantum wells without increasing the mode volume. Further, it is expected that the strong coupling could occur over the entire thickness of the Bragg structure, and the system offers an improved overlap between exciton wave function and light mode. However, advanced experiments in Bragg structures require a precise control and manipulation of quantum states of Bragg polaritons. Here, we propose and experimentally demonstrate novel methods for the modulation of Bragg polariton eigenstates. The modulation will be shown to even exceed 10 meV if the thickness of the top layer of the ZnSe-based Bragg structure is changed or if a thin silver layer is deposited on top of the structure. The Q value of the Bragg mode will be enhanced by a factor of 2.3 for a 30 nm silver layer. In addition, we report on the observation of nonlinear emission of the lower Bragg polariton mode in the hybrid structure being achieved when excitation dependent measurements are performed. Our results open the door to create a confined Bragg polariton system similar to conventional microcavities.

## Introduction

Cavity-exciton polaritons are quasiparticles resulting from the strong coupling of quantum well (QW) excitons with confined photons in a semiconductor microcavity (MC). The hybrid nature of this part light and part matter quasiparticles leads to many remarkable demonstrations such as polariton condensation and lasing^1–6^, parametric amplification^7, 8^, superfluidity^9^ and bistability^10, 11^. Since many publications on different aspects of exciton-polaritons have been published up to now, we want to refer only to some of the review articles, like refs 12–15 because of the lack of space. The wide-bandgap semiconductor-based MCs are highly beneficial in order to realize polaritonic devices operating at elevated temperatures. This is confirmed by the observation of strong coupling and polariton lasing in GaN and ZnO MCs at room temperature^16–21^. ZnSe-based MCs are particularly well suited for the investigation of cavity-exciton polaritons in semiconductors since they possess large exciton binding energies and high exciton oscillator strengths. In addition, they offer a high crystalline quality as well as dopability being beneficial for device structures. The strength of the light-matter coupling in microcavities is generally quantified in units of the Rabi energy. For this material system a promising Rabi-splitting energy of about 19 meV for a MC with 3 QWs^22^ as well as polariton lasing^23^ up to 240 K^24^ have already been reported. A large number of QWs can be used to enhance the Rabi-splitting energy since the Rabi-splitting energy increases with the square root of the number of QWs that can be positioned at the antinodes of the electric field inside the microcavity. However, in this case a thicker cavity is required which results in a larger mode volume restricting the enhancement of the Rabi-splitting energy.

In recent years, Bragg polariton structures have attracted much attention as an innovative system for the investigation of light-matter interaction^25–30^. The Bragg mode (BM) is utilized as the photonic mode and a large number of QWs can be periodically placed at the field maxima of the BM without increasing the mode volume. In this unfolded MC the Bragg polaritons arise from the strong coherent coupling between the Bragg photons and the QW excitons. Although the Bragg modes possess a smaller quality factor in comparison to a conventional MC, these polaritons possess an extremely small effective mass in comparison to those in conventional MCs^28, 31^. Since the critical temperature for polariton condensation is inversely proportional to the polariton effective mass, such structures are promising for the realization of high-temperature polariton condensation. An additional key features of this system is that the strong coupling can occur over the entire thickness of the Bragg structure^30^. It has been predicted theoretically that the peculiar dispersion of the Bragg polariton modes can give rise to many nonlinear phenomena, such as slow-light-enhanced nonlinear propagation and an ultra-efficient parametric scattering^28^. More recently the Bragg polariton samples were proposed for the realization of hyperbolic metamatrials by controlling the signs of effective masses of such mixed light-matter quasiparticles^27, 32^. Bragg polaritons were also observed in conventional Fabry-Perot type MCs^33–35^. However, the periodic arrangement of the excitonic resonances in a Bragg structure provides a maximum overlap between the exciton wave function and the photon mode^25^. From the technological point of view, such structures offer a reduced number of growth interfaces between epitaxial layers compared to a conventional MC structure which ensures a positive impact on the crystal quality of the sample^25, 26^.

In order to fully utilize the unfolded MC system as a versatile experimental platform based on Bragg polaritons some essential requirements need to be fulfilled such as the control and the manipulation of Bragg polariton eigenstates and the realization of a confined Bragg polariton system. The importance of laterally confined polaritons has been demonstrated by various potential applications^36–40^. In the conventional MC the confinement of the cavity polaritons is achieved via their excitonic as well as their photonic components. The respective confinement has been realized using surface acoustic waves^41^, local strain^2^, an exciton reservoir^42^, photonic disorder traps^43^, cavity thickness modulation^44, 45^, metal layers^46, 47^, subwavelength gratings^48^, and pillar cavities^36^. A recent review on trapped polaritons can be found in ref. 49. However, to date, mode confinement and Bragg polariton manipulation for the unfolded MCs have not been reported.

In this work, we demonstrate two promising methods to manipulate the Bragg polariton eigenstates by the variation of the thickness of the top layer of the Bragg structure or by the deposition of an Ag layer. In both cases a shift of several meV of the lower Bragg polariton (LBP) is realized. Such a large shift is crucial in order to create a potential trap for the Bragg polaritons. In addition, we will report on the nonlinear emission from the hybrid Ag-Bragg structures by performing excitation dependent photoluminescence measurements. Our calculation reveals that the Q factor of the BM can be enhanced by a factor of 2.3 by the employment of a 30 nm of Ag layer. Such an improvement of the Q factor is very important in order to increase the polariton lifetime such that the polariton relaxation to the ground state becomes faster than its radiative decay.

## Results

**Figure 1:**
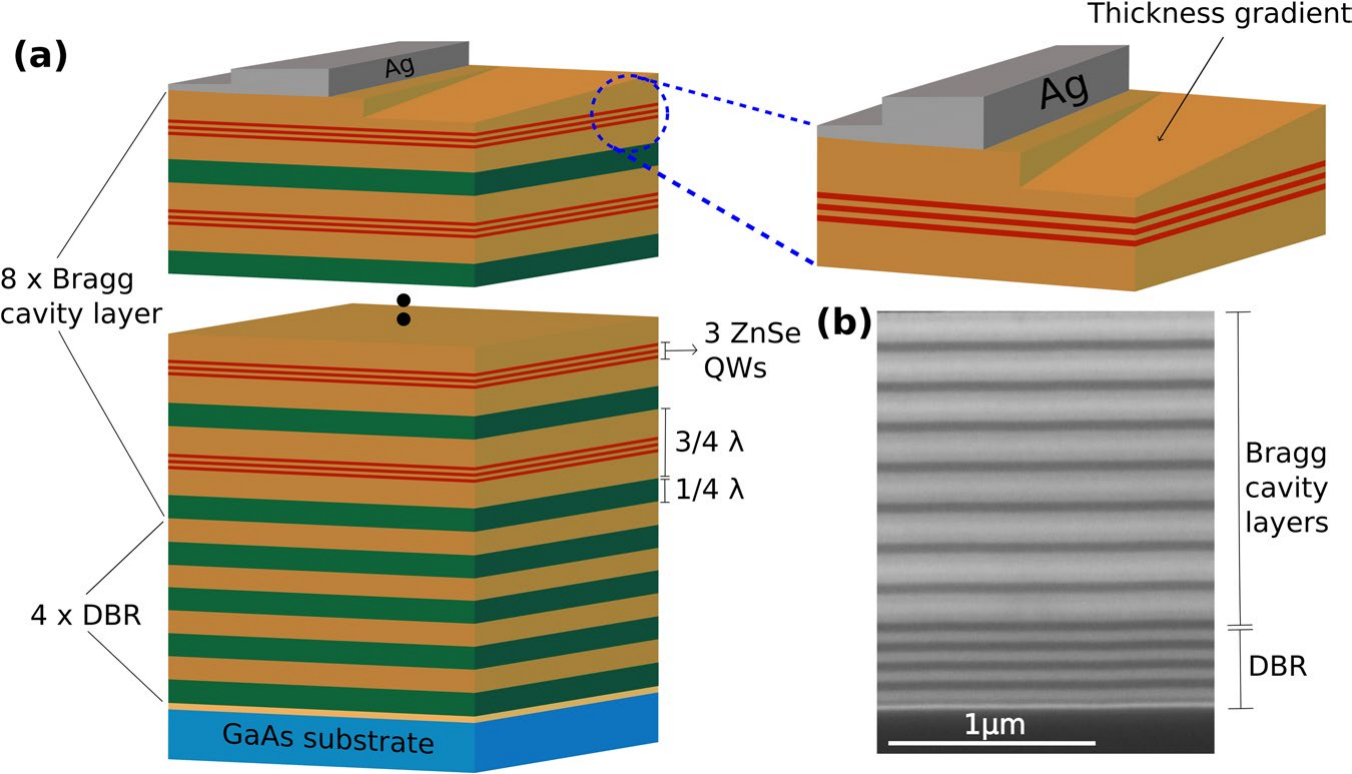
(**a**) Schematic of the investigated Bragg structure. The sample possesses a thickness gradient for the top layer as well as an Ag layer on an area without gradient. (**b**) Scanning electron microscope image of the as-grown Bragg polariton sample.

**Figure 2:**
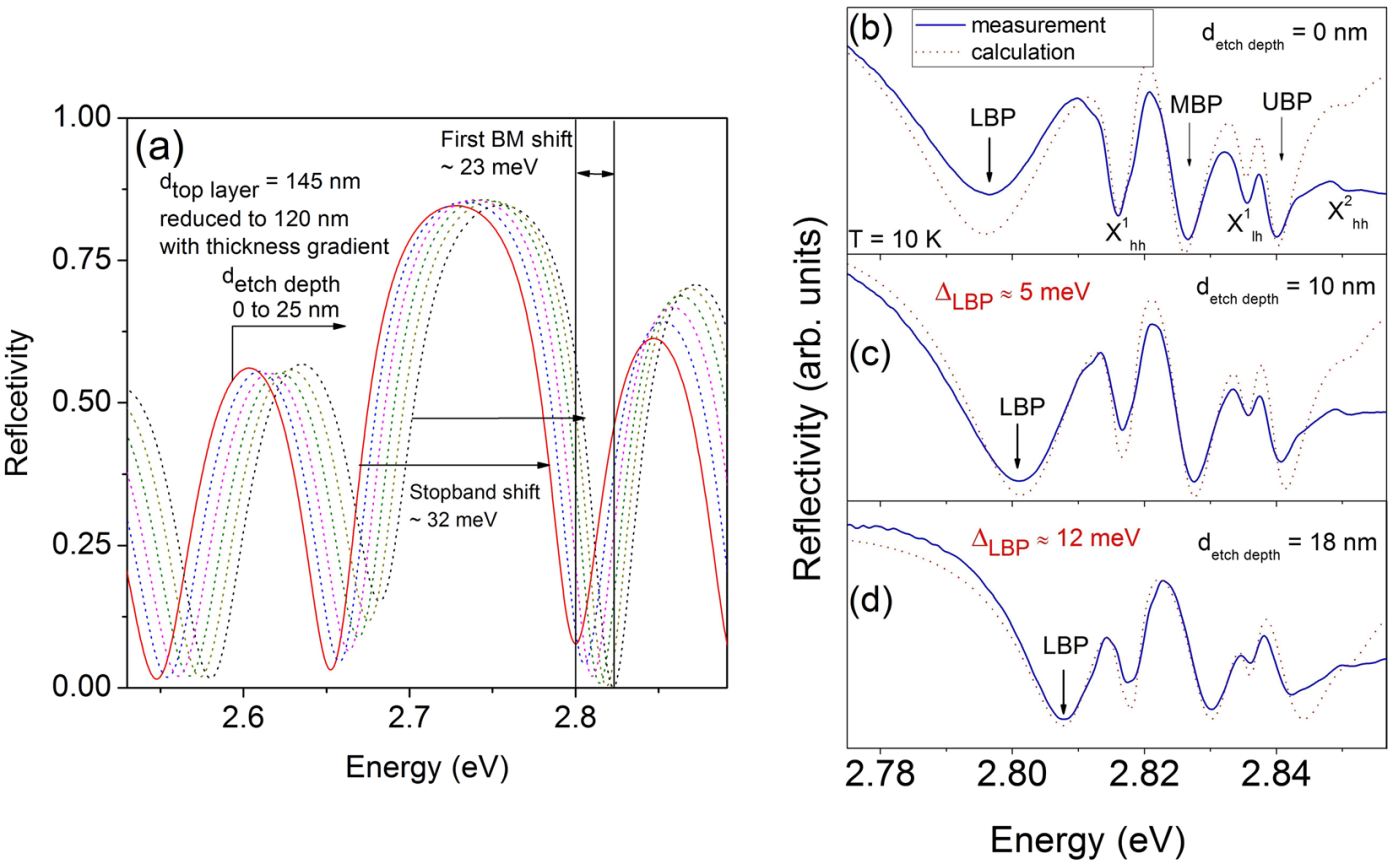
(**a**) Calculated reflectivity spectra of the cold Bragg structure obtained when the thickness of the upper layer is continuously reduced for moving along the thickness gradient, eventually ending at a 25 nm reduction achieved at the leading edge of the structure (see Fig. 1(a)). (**b**–**d**) Region of interest of the measured microreflectivity spectra (solid lines) of the Bragg sample at T = 10 K for different top layer thicknesses in comparison to the calculated spectra (dotted lines). Δ_*LBP*_ indicates the shift of the LBP mode.

Figure 1(a) shows the schematic drawing of the investigated Bragg structure. The sample consists of eight pairs of the Bragg cavity layers which were grown on four pairs of distributed Bragg reflectors (DBR). A cross-section scanning electron microscope (SEM) image of the sample is depicted in Fig. 1(b). The Bragg cavity layer is made of a 3λ/4 high- and a *λ*/4 low-index layer each. Three ZnSe QWs are inserted in each of these 3λ/4 high-index layer. The adjustment of the spectral position of the BM and the QW emission is challenging in comparison to a conventional MC, since it can only be controlled in an indirect way during epitaxial growth. Nevertheless, with such a design of the Bragg structure an optimum overlap of the electric field of the first Bragg mode and the QWs can be achieved^26^. In order to tune the Bragg mode two modifications were applied to the sample. As a first modification, a thickness gradient of the top layer was created (see methods). As a second modification, different thickness (15 and 30 nm) of Ag layer of was deposited on top of the as-grown sample as shown in Fig. 1(a). The calculated reflectivity spectrum of the periodic cold Bragg structure (without QWs) is shown in Fig. 2(a) (solid line). The photonic stop-band width is 110 meV and the position of the first BM on the high energy side can be identified at 2.799 eV. The change in the reflectivity spectrum by reducing the top layer thickness of the Bragg structure up to a maximum reduction value of 25 nm is depicted in Fig. 2(a) (dotted lines). The calculated spectra show that the photonic stop-band as well as the Bragg modes shift to higher energies by reducing the top layer thickness. A reduction of 25 nm thickness of the top layer results in a shift of about 23 meV of the first BM to higher energies. Such a large change of the spectral position of the BM is very promising with respect to the realization of an easy tuning method for the photonic component of the Bragg polaritons.

Now we will discuss the formation of the Bragg polaritons in our device structure. Figure 2(b) shows the microreflectivity spectrum of the Bragg structure at T = 10 K, where the detuning between the QW heavy-hole (${X}_{hh}^{1}$) and the first BM amounts to *δ* = *E*_*BM*_ − ${E}_{hh}^{1}$ ≈ −10 meV. Three Bragg polariton resonances are identified at the spectral positions of 2.796 eV, 2.827 eV, and 2.84 eV respectively, which we call the lower, middle, and upper Bragg-polariton (LBP, MBP, UBP). The observed three branches of the Bragg polaritons are the result of the coupling of the first BM with ${X}_{hh}^{1}$ and with the light-hole (${X}_{lh}^{1}$) excitons. The uncoupled ${X}_{hh}^{1}$ (2.817 eV) and ${X}_{lh}^{1}$ (2.835 eV) resonances are also visible in the spectrum due to the inefficient coupling of the QW exciting transitions to the BM for the upper DBR layers^25, 26^. In addition, a less pronounced reflectivity minimum at 2.851 eV can be detected which originates from the absorption of the first excited state ${X}_{hh}^{2}$. The characteristics of the reflectivity measurements can be reproduced by reflectivity calculations using transfer matrix method as shown in Fig. 2(b) (dotted line). In the calculation, the oscillator strength ratios ${X}_{lh}^{1}/{X}_{hh}^{1}=1/3$ and ${X}_{hh}^{2}/{X}_{hh}^{1}=1/8$ are implemented as well as the QW excitonic emission linewidths (FWHM = 3 meV for both ${X}_{hh}^{1}$ and ${X}_{lh}^{1}$ and FWHM = 6 meV for ${X}_{hh}^{2}$ at T = 10 K) which are taken from a reference sample. Figure 2(c,d) present the microreflectivity spectra of the same Bragg structure but for a thickness reduction of the upper layer by d_etch depth_ ≈10 nm and 18 nm, respectively, using chemical assisted ion beam etching (CAIBE). The Bragg polariton eigenstates shift to higher energies with respect to the Bragg polaritons in the unetched area of the sample (Fig. 2(b)). The change of the spectral positions of the Bragg polaritons arises from the tuning of the bare BM as shown in Fig. 2(a). The LBP will reveal shifts of Δ_*LBP*_ ≈ 5 meV and 12 meV with respect to its value obtained in the unetched area if the top layer is etched by *d*_*etchdepth*_ ≈ 10 nm and 18 nm, respectively. In addition, the linewidth of the LBP is reduced for the sample with thinner top layer due to the increase of the excitonic fraction in the Bragg polariton state. The features in the measured reflectivity spectra are in excellent agreement with those obtained from the calculations (dotted lines in Fig. 2(c,d)). Using this mode tuning technique the interaction strengths between the Bragg photons and the excitons as amounting to *ħ*Ω_*hh*_ = (25.5 ± 1) meV and *ħ*Ω_*lh*_ = (14 ± 1) meV, respectively, are derived from the measurements at 10 K for the detuning *δ* = 0. The measured values of the splitting energies are very comparable to the values of *ħ*Ω_*hh*_ = 17 meV and *ħ*Ω_*lh*_ = 12 meV, which were reported for a classical microcavity sample with three ZnSe QWs within the *λ*-cavity^47^.

**Figure 3:**
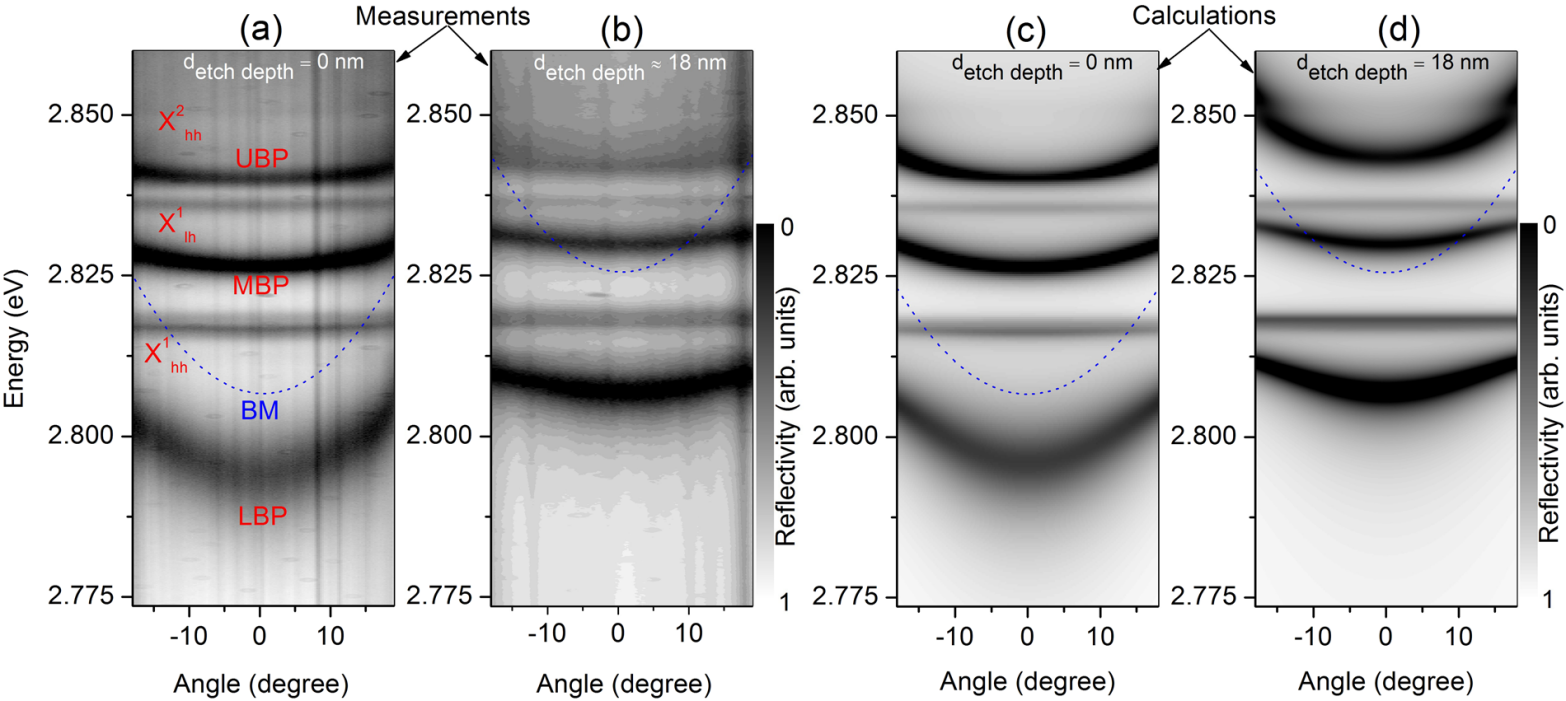
Measured (**a**,**b**) and calculated (**c**,**d**) microreflectivity intensity (arb. units) in a gray-coded map as a function of energy and angle at T = 10 K for the unetched d_etch depth_ = 0 (**a**,**c**) and etched d_etch depth_ ≈18 nm (**b**,**d**) Bragg structure. The calculated dispersion curves of the bare BM are displayed as dotted lines as well.

The existence of strong coupling in the sample can further be emphasized by performing angle-resolved measurements. Figure 3 shows the measured angle-resolved reflectivity dispersion curves of the unetched and etched area of the Bragg polariton sample at T = 10 K together with the calculated dispersion displayed in a gray-coded intensity map. The measurement reveals three branches of the Bragg polariton dispersion as well as three flat dispersion lines of the ${X}_{hh}^{1}$, ${X}_{lh}^{1}$ and ${X}_{hh}^{2}$ as shown in Fig. 3(a,b). The LBP in the unetched area of the sample is more photon-like at *θ* = 0° since the area of the sample possesses a negative detuning of *δ* $\approx$ −10 meV as depicted in Fig. 3(a). An LBP energy offset of Δ_*LBP*_ ≈ 12 meV is obtained in the sample area for which the upper layer of the Bragg structure is etched by d_etch depth_ ≈18 nm (see Fig. 3(b)). In this case the characteristic curvature of the LBP dispersion curve exhibits a more excitonic nature with a smaller curvature due to a positive detuning of *δ* ≈ +8 meV. The LBP is mostly affected in its spectral position when the detuning is changed due to the predominantly photonic nature. In addition, a weakening of the absorption and spectral broadening is observed for the UBP for positive detuning, which is caused by the overlap of the UBP with the excitonic scattering states^50^. The calculated microreflectivity intensity shown in Fig. 3 for the unetched (c) and etched (d) sample reproduces the experimental findings, except for the UBP, since the exciton scattering states were not taken into account for the calculations. The striking feature of this investigation is the controllable tuning of the LBP mode by changing a few nanometer thickness of the upper layer. The shifts of about 18 meV of the bare Bragg photon mode and of about 12 meV of the LBP resonance are quite large for an etch depth of about 18 nm. This observation is similar to the findings for a conventional MC system, where a potential depth of 9 meV was obtained^44, 51^. In this case the confinement of the photonic component is realized by etching the cavity layer 6 nm deep into the mesa structure before growing the top mirror. Likewise, the engineering of the upper layer of the Bragg structure can provide larger confinement potential for the Bragg polariton system.

**Figure 4:**
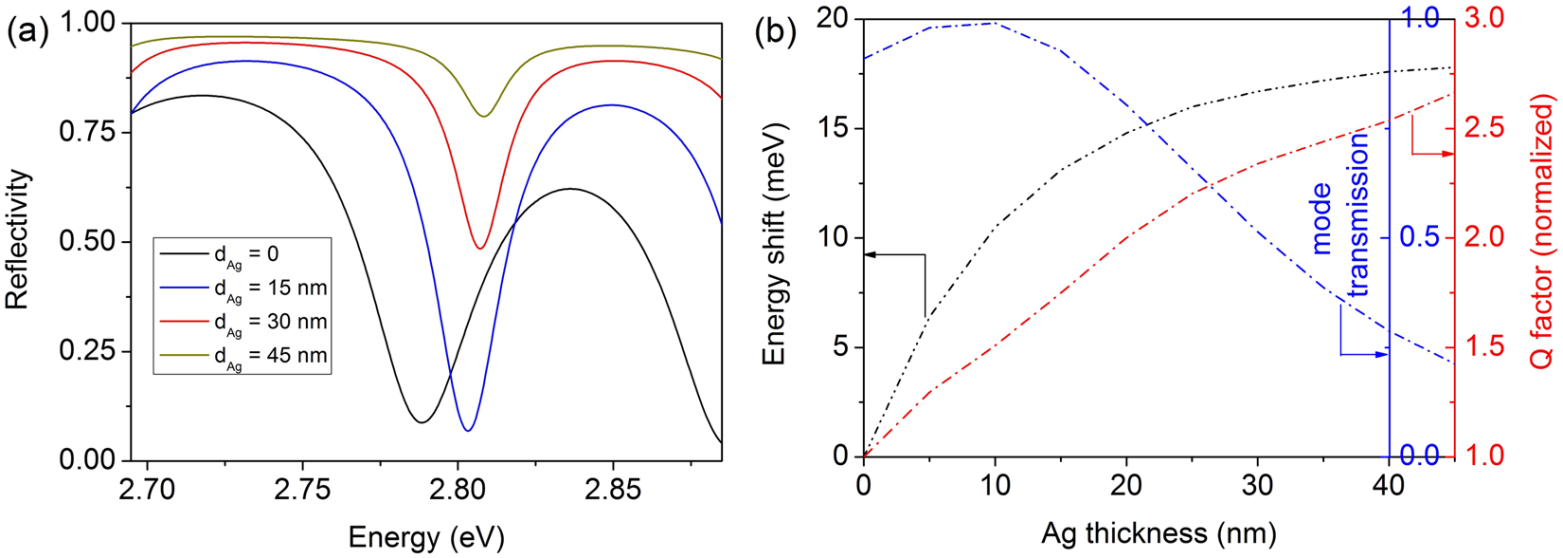
(**a**) Calculated reflectivity spectra of the cold Bragg structure in the vicinity of the first BM for different thicknesses of the Ag layer. (**b**) Calculated spectral position (black), the Q factor (red), and the mode transmission (blue dash-dotted line) of the first BM as a function of the thickness of the Ag layer.

We will now explore the influence of a thin metal layer on the optical properties of the Bragg structure. Figure 4(a) represents the relevant calculated reflectivity spectra around the first BM of the cold Bragg structure as a function of the Ag layer thickness. If the Ag thickness is zero the spectral position of the first BM is at 2.788 eV and the peak reflectivity will be close to 83.5%. The BM shifts to higher energies with increasing Ag thickness, and the peak reflectivity is increased up to 97% for an Ag thickness of 45 nm. In Fig. 4(b) the variation of the spectral position, the Q factor, and the mode transmission of the first BM as function of the Ag layer thickness are displayed. The spectral shift of the BM relative to its position in the uncovered area is quite pronounced when increasing the Ag thickness up to 30 nm and reaches its maximum value of about 18 meV for an Ag thickness of ~45–50 nm. Although the strong coupling can be achieved in these structures, the Q factor of the BM is rather low being in the order of 100. However, it can be enhanced by a factor of about 2.5 for an Ag thickness of 40 nm but it should be noted that the transmission of the Bragg resonance diminishes by increasing the thickness of the Ag layer (Fig. 4(b) (blue dot-dashed line)). Hence we need an optimized Ag thickness in order to get a high transmission and Q factor as well as a pronounced spectral shift at the same time. The theoretical calculation indicates that for an Ag thickness of 30–35 nm an optimum trade-off for all these requirements is reached and the calculated E-field distribution for the coupled mode within the periodic structure shows a reduction by a factor of 0.7 only in comparison to the uncovered sample.

**Figure 5:**
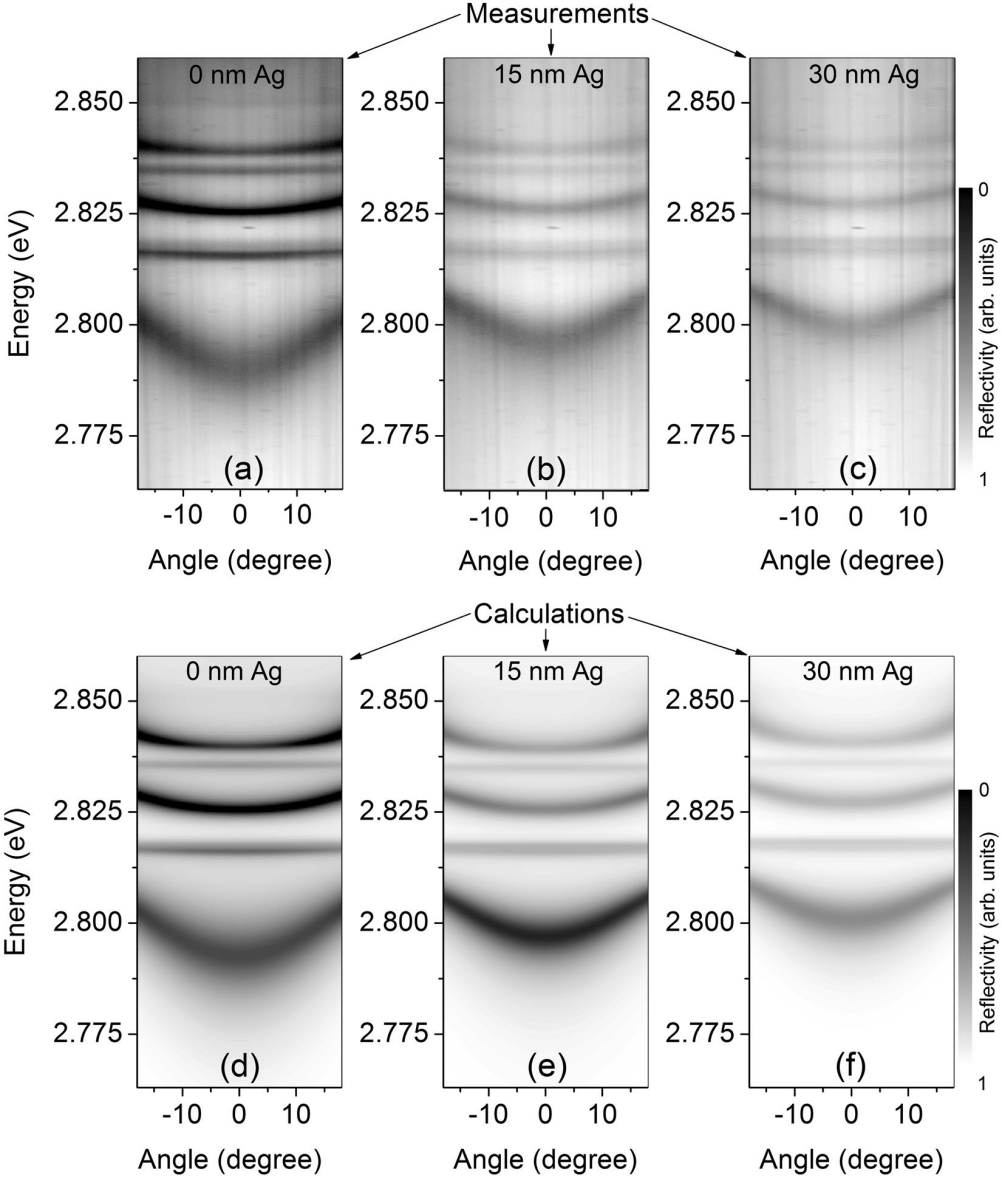
(**a**–**c**) Angle resolved microreflectivity measurement of the Bragg polariton dispersion for different thicknesses of the Ag layer. The experimental results are compared to the calculated dispersions (**d**–**f**).

The angle-resolved reflectivity measurements of the Bragg polariton sample with and without Ag layer at 10 K are shown in Fig. 5 and compared to the calculated dispersion. The investigated area of the sample possesses a negative detuning of *δ* ≈ −14.5 meV (without Ag) and the spectral position of the LBP mode (*θ* = 0°) can be identified at 2.791 eV (see Fig. 5(a)). After deposition of an Ag layer on top of the Bragg structure the Bragg polariton eigenstates shift to higher energies. Beside the reduction of the absorption contrast, the linewidth of the LBP resonance decreases with increasing Ag thickness and for the UBP no spectral widening is observed. The reason of such a linewidth narrowing is due to the enhancement of the Q factor of the photonic component as shown in Fig. 4(a), which can compensate the absorption losses originating from the overlap between the UBP and the excitonic scattering states. A shift of about 6.0 meV (9.5 meV) of the LBP mode [*θ* = 0°] compared to its position in the metal-free sample is induced by a 15 nm (30 nm) thick Ag layer, respectively (Fig. 5(b,c)). The experimental observations are in very good agreement with the calculations as presented in Fig. 5(d–f). The observed spectral shift exceeds the value in the order of 0.1–1 meV which was reported for conventional MCs^49^. Hence, this metal induced spectral shift could be utilized as a lateral trapping potential for the Bragg polaritons when using a structured metal layer such as gratings or disks on top of the unfolded MC structure.

**Figure 6:**
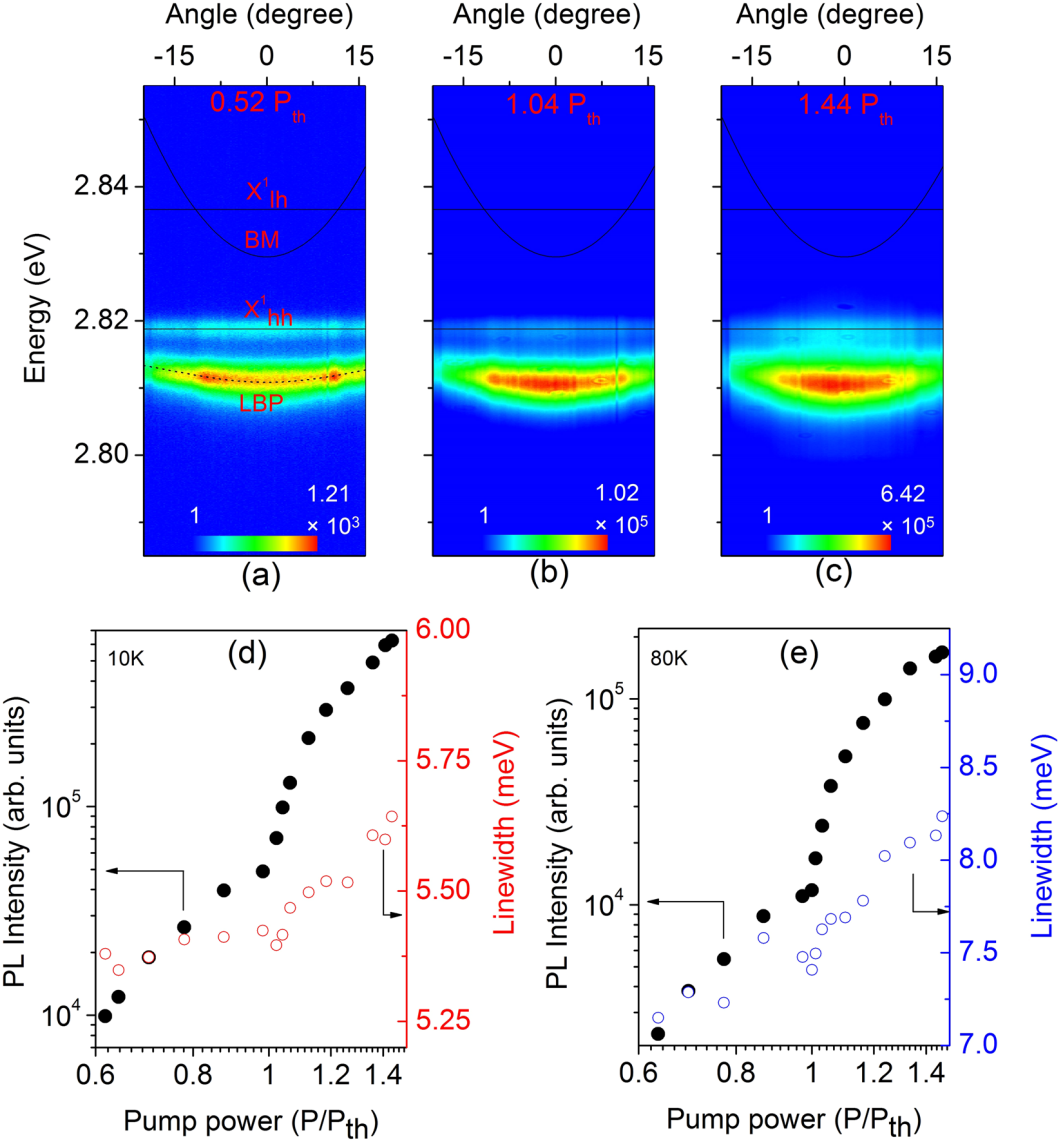
(**a**–**c**) The angular-resolved PL spectra of the hybrid Ag-Bragg structure for three different excitation powers at T = 10 K, the calculated bare BM (solid line) and LBP (dotted line) dispersion are displayed as well. The PL intensity and linewidth of the LBP mode (*θ* = 0°) as a function of the excitation density for T = 10 K (**d**) and T = 80 K (**e**) are plotted.

Up to now, we have demonstrated the influence of the Ag layer on the Bragg polariton eigenstates. In the next step we investigate the properties of Bragg polariton photoluminescence (PL) from the hybrid Ag-Bragg structure. The angular-resolved emission spectra of the Bragg polaritons at a temperature of T = 10 K are shown in Fig. 6(a–c) for different excitation densities. The sample contains a 30 nm thick Ag layer and the measurements were performed at a positive detuning of *δ* ≈ +11.0 meV. At low excitation densities the LBP dispersion branch is observed which possesses a little stronger PL intensity at higher angles (see Fig. 6(a)). In addition, the uncoupled ${X}_{hh}^{1}$ emission can be identified. With an increase of the excitation power (P = 1.04 P_th_, P_th_ is the nonlinear threshold), the emission of the LBP mode exhibits a smooth intensity distribution around *θ* = 0° due to the enhancement of polariton-polariton scattering. By further increasing the excitation power (P = 1.44 P_th_) a slight shrinkage of the momentum space distribution can be detected, as shown in Fig. 6(c), and the LBP emission is broadened. Figure 6(d) presents the PL intensity and linewidth of the LBP (*θ* = 0°) as a function of the excitation density at T = 10 K. A clear nonlinear increase of the emission intensity is observed for the LBP mode above P = P_th_ indicating a threshold like behavior. Below threshold, the spectral width of the LBP emission broadens with increasing density; however, at the threshold, the linewidth drops slightly then again starts to increase with the excitation power due to decoherence induced by polariton-polariton interaction and heating^52, 53^. A very similar nonlinear behavior of the LBP mode is observed at a temperature of T = 80 K (Fig. 6(e)). Due to the spectral position and dispersion it is clear that the nonlinear emission comes from a polaritonic mode in strong coupling regime. This nonlinear emission behavior of the LBP mode is also observed for a small positive detuning of *δ* ≈ +3.0 meV at 10 K (see Supplementary information). However, a pronounced bottleneck effect can be detected for negative detunings (Fig. S2) which hinders the observation of nonlinear emission in this case. For negative detunings the photonic fraction of the LBP mode is considerably increased which results in a reduction of the polariton lifetime. Hence, photon like LBPs radiatively decay during the relaxation near the bottleneck before reaching the ground state of the polariton dispersion. For positive detunings, the polariton lifetime is long enough so that they can reach the energy minimum of the polariton dispersion. More details on the kinetics and thermodynamics of polaritons in MCs are discussed in refs 54–57.

**Figure 7:**
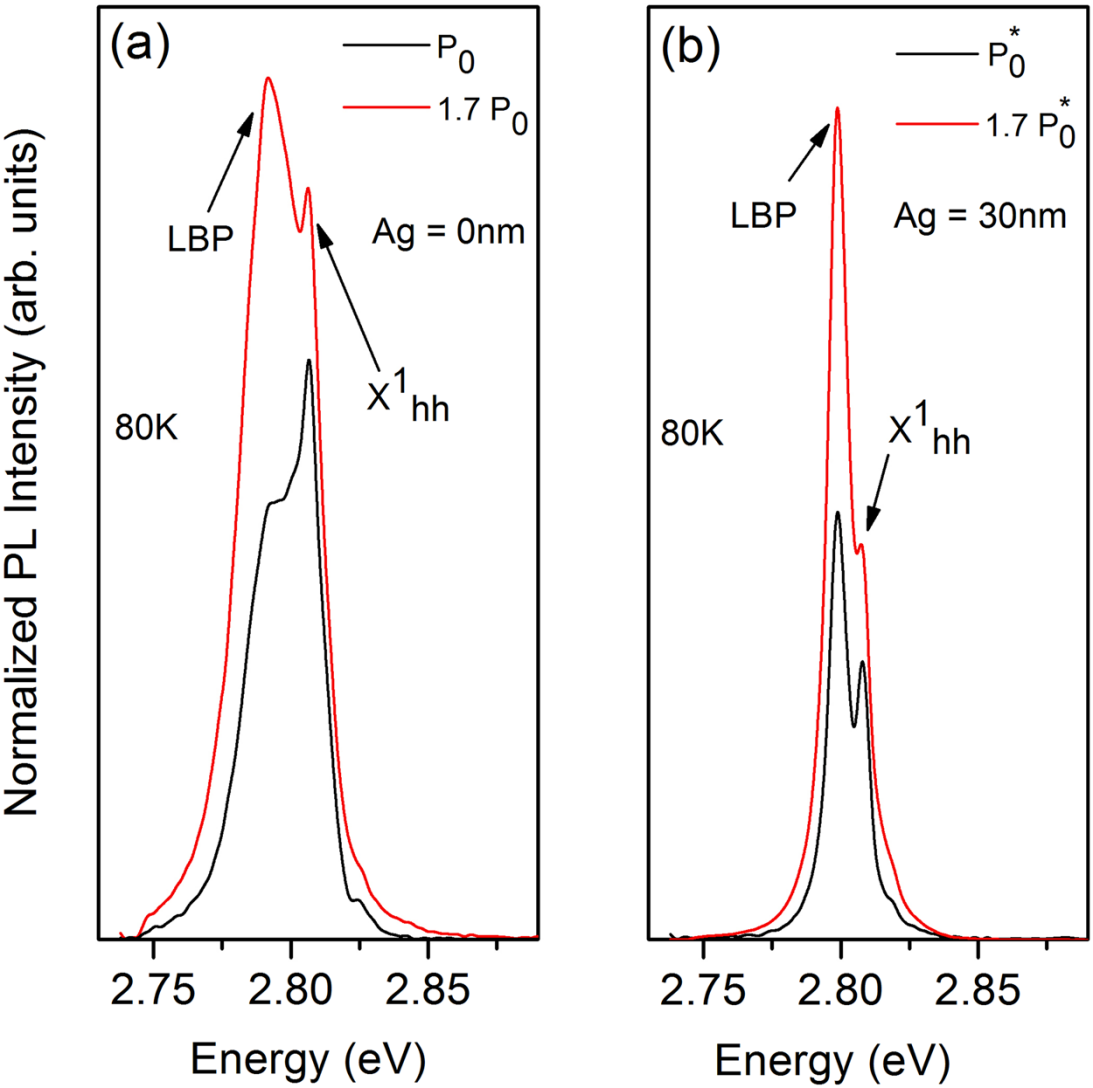
Emission spectra (*θ* = 0°) of the unfolded MC structure without Ag (**a**) and with Ag (**b**) for different excitation powers at T = 80 K.

In our Bragg polariton system, a more efficient stimulated scattering is observed for the positive detuning. However, in the case of nonlinear emission the linewidth narrowing at threshold is not significant. Furthermore, we have not observed a spectral blue shift of the LBP energy with increasing excitation power, which typically arises in a conventional MC due to phase space filling of the excitons and exciton-exciton interactions^3, 58^. Above threshold a small red shift of the LBP by about 0.3 meV can be detected (Fig. 6(c)) attributed to a local heating effect of the sample which is confirmed by the spectral shift of the uncoupled ${X}_{hh}^{1}$ energy in the real space PL measurement. Hence, before stating a Bragg polariton lasing effect further evidence for this would have to be given by measuring the coherence properties of the LBP emission^1^. Nevertheless, a significant improvement of the LBP emission has been observed after deposition of an Ag layer on the Bragg structure. Figure 7 represents a comparison between the LBP emission from the metal free Bragg structure and the hybrid Ag-Bragg structure. At low excitation power (P = P_0_) the uncoupled ${X}_{hh}^{1}$ emission from the metal free Bragg structure is dominant compared to the emission of the LBP resonance (Fig. 7(a)). When the pump power is increased to P = 1.7 P_0_ the emission of the LBP mode exceeds the ${X}_{hh}^{1}$ emission. In the case of the hybrid Bragg structure, a strong emission of the LBP mode can be observed for both low and high excitation powers (${{\rm{P}}}_{0}^{\ast }$ and 1.7 ${{\rm{P}}}_{0}^{\ast }$) as shown in Fig. 7(b). In addition, the LBP emission linewidth in the hybrid structure is much narrower in comparison to the Bragg polariton in the metal-free structure owing to the enhancement of the vertical photon confinement.

Indeed the deposition of an Ag layer on top of the Bragg structure presents several important advantages: (a) it provides an alternative technique to create a lateral confinement potential for the Bragg polariton system when using structured metal layers; (b) it is expected to increase the lifetime of the Bragg photon which is very crucial for the polariton relaxation to the ground state; (c) it offers a possibility to realize an electrical tuning of the Bragg polaritons. In order to take full advantage of the metal layer on the Bragg structure the following steps could be utilized: first, the upper layer of the Bragg structure could be engineered to produce a lateral confinement potential; afterwards a metal layer could be deposited on top of the structured upper layer. Such a hybrid metal-Bragg structure will ensure a large lateral confinement potential for the Bragg polaritons as well as a strong vertical photon confinement. It should also be noted that the presented Bragg structure has only eight Bragg polariton stacks. It has been discussed in ref. 26 that the splitting energy between the LBP and UBP can be raised to up 60 meV by doubling the number of Bragg polariton stacks and the utilization of a threefold number of Bragg pairs can provide an interaction strength of 75 meV. In addition, this hybrid system can easily be realized using a wide variety of high refractive index contrast organic or inorganic materials. Furthermore, our proposed techniques for the creation of the confined Bragg polariton system and the evidence of nonlinear emission from the Bragg structure will promote the investigation of other nonlinear phenomena such as stimulated scattering, amplification, and lasing in the Bragg polariton system.

## Discussion

We have experimentally realized two promising techniques for the modulation of the Bragg polariton eigenstates in a ZnSe-based unfolded microcavity. The Bragg polariton modes exhibit a shift to higher energies when the thickness of the upper layer of the Bragg structure is reduced or a thin Ag layer is deposited on top of the Bragg structure. Shifts of about 12 meV and 9.5 meV of the LBP mode are induced by an etch depth of about 18 nm in the upper layer and by the deposition of a 30 nm Ag layer on the unfolded MC structure, respectively. Such large shifts of the Bragg polariton are striking with respect to the realization of lateral potential traps for the Bragg polariton system. The experimental observations are in very good agreement with calculations based on the transfer matrix method. In addition, a nonlinear increase of the LBP emission intensity is achieved up to T = 80 K when the excitation intensity is increased applied to the hybrid Ag-Bragg structure. Our results will help to create a novel platform for the exploration of light-matter interactions based on confined Bragg polaritons in an unfolded microcavity system with a large number of QWs.

## Methods

The investigated sample was grown by molecular beam epitaxy. The high-index layer of the Bragg cavity layer, which includes 3 ZnSe QWs is constituted of Zn_0.79_ Mg_0.21_ S_0.23_ Se_0.77_ with a total thickness of 3λ/4 (144 nm) while the low-index layer consists of a superlattice of MgS and Zn_0.63_ Cd_0.37_ Se with a total thickness of *λ*/4 (44.7 nm). The refractive index contrast between the high- and low-index materials is in the order of Δn = 0.4. It should be noted that a large index contrast is crucial in order to realize spectrally narrow Bragg modes^25^. Further details on the growth and fabrication processes can be found in our previous report^26^. The top layer of the Bragg structure is a 3*λ*/4 high-index layer. For the first tuning method of the Bragg eigenenergy the top layer thickness was reduced by CAIBE. By using a shadow mask a thickness gradient was realized with a maximum etch depth in the top layer of about 25 nm as shown in Fig. 1(a). An Ag layer was deposited on the sample surface as an alternative tuning method for the Bragg polaritons. For this investigation, 15 nm and 30 nm of Ag layers were deposited on top of the Bragg structure by electron-beam physical vapor deposition. For optical characterizations microreflectivity and microphotoluminescence measurements were performed on different areas of the sample with and without Ag cover layer. A white light source and a Ti:sapphire laser operating in pulsed mode (82 MHz rate, excitation wavelength *λ* = 406 nm) were used as the excitation sources for the microreflectivity and microphotoluminescence measurements, respectively. The exciting laser spot diameter was about 3 *μ*m. The signals from the sample were collected through a microscope objective (collecting angles up to ±20^0^) and detected using a cooled charge-coupled device spectrometer. The experimental reflectivity spectra and dispersions were compared to calculations using the transfer matrix method (software CAMFR^59^). The parameters for the simulations are based on layer thicknesses measured by X-ray diffraction. The semiconductor refractive index dispersions were taken from ref. 60 and the Drude model was employed for the Ag layer^61^.

## Electronic supplementary material

Supplementary Material
