# Supplementary material for: Tunable Bragg polaritons and nonlinear emission from a hybrid metal-unfolded ZnSe-based microcavity
Source: Sci Rep. 2017 Apr 10;7:767. doi: 10.1038/s41598-017-00878-2 (PMC5429702; doi:10.1038/s41598-017-00878-2)
Supplement: Supplementary file 1 — Supplementary Material [file 41598_2017_878_MOESM1_ESM.pdf]

Supplementary information

Tunable Bragg polaritons and nonlinear emission from a hybrid metal-unfolded ZnSe-based microcavity

SK. Shaid-Ur Rahman[1*], Thorsten Klein[2,3], Jürgen Gutowski[1], Sebastian Klembt[2,4], and Kathrin Sebald[1]

[1]Semiconductor Optics, Institute of Solid State Physics, University of Bremen, Bremen, 28334, Germany

[2]Semiconductor Epitaxy, Institute of Solid State Physics, University of Bremen, Bremen, 28334, Germany

[3]Present address: BIAS, Bremer Institut für angewandte Strahltechnik GmbH, Klagenfurter Str. 2, 28359 Bremen, Germany

[4]Present address: Technische Physik, Universität Würzburg, Am Hubland, D-97074 Würzburg, Germany

*rahman@ifp.uni-bremen.de

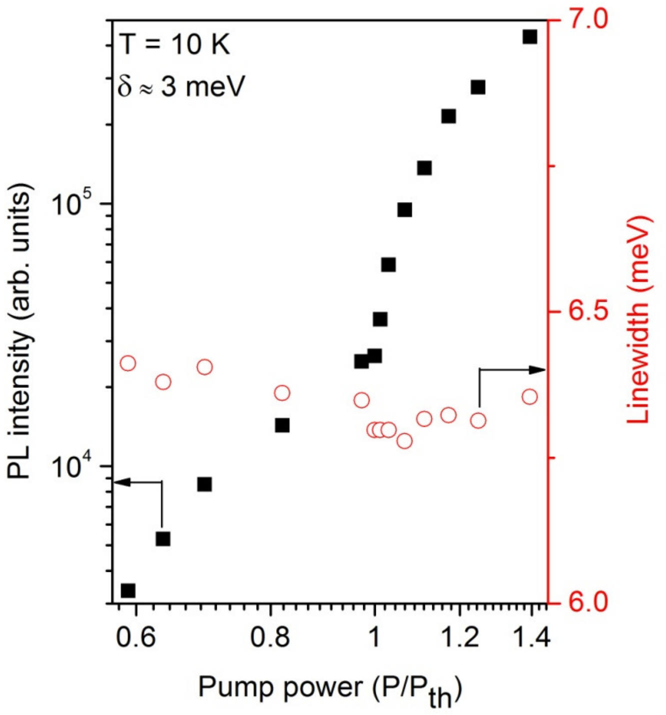

Supplementary Figure S1. The PL intensity (squared dot) and linewidth (circled dot) of the LBP mode ($\theta = 0^0$) as a function of the excitation density for a detuning of $\delta \approx 3$ meV. The measurements are performed at a temperature of T = 10 K.

Here, we present Bragg polariton emission properties for two different detunings ($\delta \approx 3$ meV and $\delta \approx -10$ meV) of hybrid Ag-Bragg structure. The sample contains a 30 nm thick Ag layer and the measurements were performed at T = 10 K.

Fig. S1 shows the PL intensity and linewidth of the LBP (at normal incidence, $\theta = 0^0$) as a function of pump power for a detuning of $\delta \approx 3$ meV. The emission behavior is similar to the one in the main manuscript (Figure 6 (d) and (e)) for the LBP mode. The spectral width of the LBP emission drops slightly at the threshold region and then starts to increase with the excitation power due to the polariton-polariton interaction.

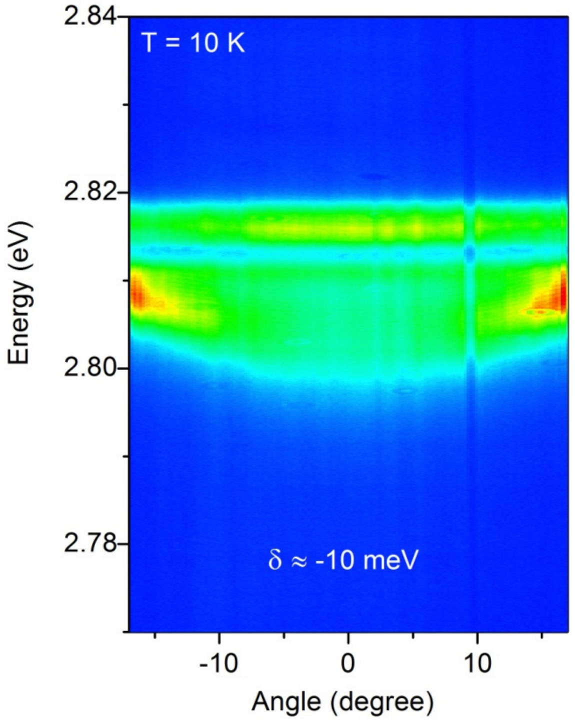

Supplementary Figure S2. The angular-resolved PL spectra of the hybrid Ag-Bragg structure for a negatively detuned (δ ≈ -10 meV) position of the sample measured at T = 10 K.

For the negative detuning, light is mostly emitted from the relaxation bottleneck region of the polariton dispersion curve as shown in Fig. S2. In this case, the polariton ground state is highly diluted and an intense population of the polariton can be found at higher angles (17 - 20^0). This implies that an inefficient polariton relaxation event occurs at negative detunings. The polariton scattering efficiency, including polariton-polariton and polariton-phonon interactions are related to the cavity-exciton detuning δ, which determines the excitonic and photonic fractions in the polariton branch[1]. The lifetime of the polaritons is mainly inversely proportional to the photonic fraction[2]. When going from positive to negative δ values the photonic fraction of the LBP mode increases so that the lifetime of the polariton decreases. At

the same time, the polariton relaxation time increases when going from positive to negative values due to the smaller excitonic fraction of the LBPs and a decreased polariton-polariton and polariton-phonon interactions. Hence at the negative detunings, the photonlike LBPs radiatively decay during the process of relaxation near the bottleneck before actually reaching the energy minimum. The relaxation kinetics owing to the onset of the polariton-polariton scattering mechanism can be further enhanced by increasing the pump power. However, as the Q factor is quite low for the investigated Bragg structure the polariton-polariton scattering event for photonlike polaritons is not rich enough to overcome the bottleneck effect. The polariton stimulated scattering is favored for the positive detuning of this hybrid Ag-Bragg structure.

Reference

(1) Levrat J., Butté R., Feltin E., Carlin J., & Grandjean N. Condensation phase diagram of cavity polaritons in GaN-based microcavities: Experiment and theory *Phys. Rev. B* **81,** 125305 (2010).

(2) Lai, Y.-Y. et al. Crossover from polariton lasing to exciton lasing in a strongly coupled ZnO microcavity. *Sci. Rep* **6,** 20581 (2016).
